# Evidence of Structural Inhomogeneities in Hard-Soft Dimeric Particles without Attractive Interactions

**DOI:** 10.3390/ma13010084

**Published:** 2019-12-23

**Authors:** Gianmarco Munaò, Franz Saija

**Affiliations:** 1 Dipartimento di Scienze Matematiche e Informatiche, Scienze Fisiche e Scienze della Terra, Università degli Studi di Messina, Viale F. Stagno d’Alcontres 31, 98166 Messina, Italy; 2 CNR-IPCF, Viale F. Stagno d’Alcontres 37, 98158 Messina, Italy

**Keywords:** Monte Carlo simulations, soft matter, aggregation, integral equations, clusters

## Abstract

We perform Monte Carlo simulations of a simple hard-soft dimeric model constituted by two tangent spheres experiencing different interactions. Specifically, two hard spheres belonging to different dimers interact via a bare hard-core repulsion, whereas two soft spheres experience a softly repulsive Hertzian interaction. The cross correlations are soft as well. By exploring a wide range of temperatures and densities we investigate the capability of this model to document the existence of structural inhomogeneities indicating the possible onset of aggregates, even if no attraction is set. The fluid phase behavior is studied by analyzing structural and thermodynamical properties of the observed structures, in particular by computing radial distribution functions, structure factors and cluster size distributions. The numerical results are supported by integral equation theories of molecular liquids which allow for a finer and faster spanning of the temperature-density diagram. Our results may serve as a framework for a more systematic investigation of self-assembled structures of functionalized hard-soft dimers able to aggregate in a variety of structures widely oberved in colloidal dispersion.

## 1. Introduction

The formation of aggregates in systems composed by identical building blocks is a process of paramount interest in different fields of physical, chemistry, biology and material science [1,2]. The spontaneous appearance of such structures under appropriate external conditions is observed in a large variety of systems, including colloidal suspensions [3,4], polymer nanocomposites [5,6,7], proteins [8,9], surfactants [10,11] and block copolymers [12,13,14]. In almost all theoretical and computational models envisaged for studying these systems, the appearance of complex structures is closely related to the attractive interactions between the building blocks. In this context, many different protocols have been proposed in order to describe the formation of aggregates by using simple models. One of the most successfull is based on patchy particles [15], which have been extensively studied by both experimental [16,17,18,19] and computer simulation [20,21,22] approaches. According to such models, a single building block is usually considered as an hard sphere whose surface is decorated with a variable number of attractive sites (called patches) which lead the formation of aggregates through the self-assembly mechanism. This approach, well suited to study the formation of aggregates in surfactants and proteins, has been extended also to dimeric systems, hence giving rise to patchy dimers [23,24]. The possibility to tune the aspect ratio as well as the size of the monomeric units (usually simple spheresl) constituting the dimer allows to explore a large variety of conditions, from the cluster formation to the gas-liquid phase separation. Recently, experiments [25] and computer simulation studies [26] have shown that patchy dimers can be succesfully used also to encapsulate spherical target particles. According to the above mentioned models for patchy dimers, all the monomeric units experience a steric interaction except a pair of monomers, belonging to different dimers, which interact via an attractive potential, in some cases modulated by an angular-dependent parameter. Such a scheme shows the double advantage to be very simple to implement and to give rise to a large collection of self-assembled aggregates. The thermodynamic parameters which usually play a significant role in this process are the temperature and the density of the system: a proper combination of both of them may led to the desired structure.

More recently, also the role played by the softness of the interaction potential has been gaining a remarkable importance [27], since the traditional representation of colloids as hard spheres can be seen as an extreme case of more realistic soft colloids in which the degree of softness is very low. The existence of a softness in the interaction potential has remarakble effects on the final properties of the systems: for instance, it has been shown that softly-interacting fluids may exhibit reentrant melting or waterlike anomalies [28,29,30]. In addition, these systems usually freeze in a large variety of crystalline phases [31]. Finally, soft colloids have been used also as models for microgels [32,33,34,35,36], catching the swelling-deswelling transition experimentally observed in these systems. However, the role played by the softness in the self-assembly process has been less extensively investigated. In particular, even if simulation studies of soft self-assembling systems have been performed [37,38] the appearance of aggregates is due to both the attraction strength and the softness of the potential. As a consequence, a proper understanding of the role played by the softness alone is not simple to deduce.

In the present work we aim to shed light on this topic by studying the possible onset of aggregates in a dimeric system which does not exhibit any attractive interaction. This choice is also proposed in order to mimick the behavior of colloidal systems in which the attractive contributions are negligible in comparison to steric effects, as can be expected in case of large colloidal molecules and small amount of depletant [39]. In our approach a building block is composed by two tangent spheres which experience different interactions and are rigidly connected in a dimeric configuration. A pair of hard spheres belonging to different dimers interact via a hard core repulsion, while the interactions between the soft spheres are modeled by using the Hertzian potential, which belong to the class of bounded softly repulsive potentials. The cross correlations are also modeled by the Hertzian potential, in which the degree of softness is lower. The onset of aggregates in hard-soft dimeric particles has been already experimentally observed [40,41,42] even if in the experiments a neat attraction between the soft monomers was present. Here we investigate the formation of aggregates by using Monte Carlo (MC) simulations and integral equation theories of molecular liquids. The fluid structure of hard-soft dimers is characterized by computing radial distribution functions and structure factors; the onset of aggregates is analyzed by calculating bond distributions, average cluster sizes and cluster size distributions. The integral equation approach is based on the Reference Interaction Site Model (RISM) theory of the fluid phase [43]: this method has been successfully implemented to describe, among others, structure and thermodynamics of dimeric fluids [44,45], aggregation in colloidal particles [46,47], self-associating liquids [48,49,50] and their mixtures [51,52]. The implementation of the RISM scheme is particularly indicated for studying the model at issue, since it allows us to quickly scan the low-temperature regime where the onset of aggregates is expected and where MC simulations typically require long times before attaining the equilibration.

## 2. Models and Methods

### 2.1. The Intermolecular Potential

**Figure 1:**
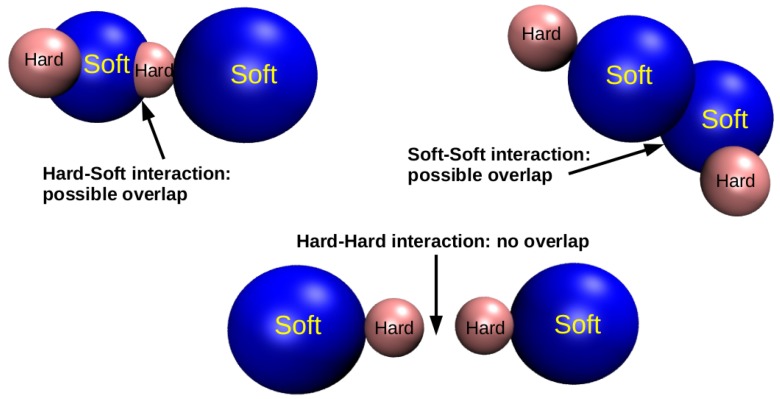
Models of hard-soft dimers investigated in this work and their possible interactions. The blue and pink colors label soft and hard monomers, respectively.

In our model a hard-soft dimer is constituted by two tangent spheres of sizes $\sigma_{1}$ and $\sigma_{2}$ rigidly bonded together. By labeling with 1 and 2 the two monomers, we set three different kinds of site-site interactions:

(1) $$\begin{array}{c} \;V_{11}\left(r\right)=\left\{\begin{array}{cc} \epsilon {\left(1-r/\sigma_{1}\right)}^{\alpha } & \;\mathrm{if}\;r\leq \sigma_{1}\\ 0 & \;\mathrm{if}\;r>\sigma_{1} \end{array}\right., \end{array}$$

(2) $$\begin{array}{c} \;V_{12}\left(r\right)=\left\{\begin{array}{cc} \epsilon {\left(1-r/\sigma_{12}\right)}^{\delta } & \;\mathrm{if}\;r\leq \sigma_{12}\\ 0 & \;\mathrm{if}\;r>\sigma_{12} \end{array}\right., \end{array}$$

(3) $$\begin{array}{c} \;V_{22}\left(r\right)=\left\{\begin{array}{cc} \infty & \;\mathrm{if}\;r\leq \sigma_{2}\\ 0 & \;\mathrm{if}\;r>\sigma_{2} \end{array}\right., \end{array}$$

where *r* is the interparticle distance and $\sigma_{12}=\left(\sigma_{1}+\sigma_{2}\right)/2$. In what follows, ϵ and $\sigma_{1}$ are assumed as units of energy and length, respectively. In all cases we have set $\sigma_{2}=\sigma_{1}/2$ in order to emphasize the role played by the soft interactions in driving the formation of local inhomogeneities. According to the Equation (1) two soft monomers interact through the Hertzian potential with exponent α=5/2, following previous studies of Hertzian spheres [31,53,54]. The interaction between hard monomers is represented by a bare hard-core potential, modelled through the Equation (3). Finally, the hard-soft interaction is given by the Hertzian potential with exponent δ=7/2 (Equation (2)), therefore slightly more repulsive than the soft-soft interaction. We shall henceforth make use of reduced units for pressure, temperature and number density, by setting $P^{*}\equiv P\sigma^{3}/\epsilon$, $T^{*}\equiv k_{B}T/\epsilon$ and $\rho^{*}\equiv \rho \sigma^{3}$. For simplicity, afterwards we will omit the asterisks in the notation of the reduced units. A schematic representation of the hard-soft dimer model is given in Figure 1: a pair of dimers can partially overlap if two soft monomers (depicted in blue) or a hard (depicted in pink) and a soft monomers come in close contact. Conversely, two hard monomers can not overlap, since they experience only a hard-core repulsion.

### 2.2. Monte Carlo Simulations

We have systematically performed MC simulations in order to check for the onset of local inhomogeneities which may suggest the existence of aggregated structures. For such an aim we have typically simulated 500 hard-soft dimers in the NVT ensemble by using the Metropolis algorithm and encompassing a wide range of temperatures and densities. A single MC cycle is realized by performing *N* trial single-dimer moves, *N* indicating the total number of dimers. Each of these trial moves is a random choice between a center-of-mass translation and a rotation around a coordinate axis. The acceptance rule is designed in oder to satisfy the detailed balance. Also, the maximum random shift and rotation are systematically adjusted along the simulation run in order to keep the ratio of accepted to total number of moves close to 50%. We have verified that this ratio holds even at the lowest temperature investigated, namely $T=1\times 10^{-3}$. We have also checked the importance of size effects, comparing in various cases results obtained with 500 and 2048 particles, without finding any significant difference. All simulations have been performed in a cubic simulation box with periodic boundary conditions. For $T\geq 1\times 10^{-2}$ the convergence has been obtained by performing $2\times 10^{5}$ MC sweeps followed by the same number of sweeps in the production stage. For lower temperatures we have first performed $2\times 10^{6}$ MC sweeps, then averaging the structural and thermodynamic properties over a subsequent set of 10${}^{6}$ MC sweeps. In particular, the radial distribution function g(r) has been computed every 10${}^{4}$ MC sweeps in the production stage. Cluster size distributions have been mediated over the last 500 configurations in the production stage. Standard deviations have been calculated in the production run according to the formula:

(4) $$s=\sqrt{\frac{\sum_{i=1}^{N}{\left(f_{i}-\bar{f}\right)}^{2}}{\left(N_{s}-1\right)}}$$

where $f_{i}$ and $\bar{f}$ are respectively the instantaneous and the average value of a given function and $N_{s}$ is the number of simulation time steps. If not explicitly reported in the figures, error bars indicating the standard deviations are smaller than symbol sizes of the corresponding curves.

### 2.3. Integral Equation Theories

In order to perform a fast screen of the low-temperature regime, beside the MC simulation we have also investigated structural and thermodynamic properties of the hard-soft dimers by employing the RISM framework [43]. According to this approach the structure of a diatomic fluid is characterized by a set of four site-site intermolecular pair correlation functions $h_{ij}\left(r\right)$ where (i,j)=(1,2) These functions are related to the intermolecular direct correlation functions $c_{ij}\left(r\right)$ (which, in turn, are related to the interaction potential) by a matrix generalization of the Ornstein-Zernike equation for simple fluids [55]. This equation is usually written in the *k*-space as:

(5) H(k)=W(k)C(k)W(k)+ρW(k)C(k)H(k)

where $H\equiv [h_{ij}\left(k\right)]$, $C\equiv [c_{ij}\left(k\right)]$, and $W\equiv [w_{ij}\left(k\right)]$ are 2×2 symmetric matrices. The matrix elements $w_{ij}\left(k\right)$ are obtained by Fourier transforming the intramolecular correlation functions, which read as:

(6) $$w_{ij}\left(k\right)=\frac{\sin[kL_{ij}]}{kL_{ij}}\;,$$

$L_{ij}$ is the bond length, given either by $L_{ij}=\sigma$, if i≠j, or by $L_{ij}=0$, otherwise. In this work Equation (5) is solved by adopting the hypernetted chain (HNC) expression [55]:

(7) $$c_{ij}\left(r\right)=\exp\left[-\beta V_{ij}\left(r\right)+\gamma_{ij}\left(r\right)\right]-\gamma_{ij}\left(r\right)-1$$

where $\beta =1/T^{*}$ and $\gamma_{ij}\left(r\right)=h_{ij}\left(r\right)-c_{ij}\left(r\right)$. We have chosen this closure approximation because it provides excellent results when applied to softly interacting systems, such as the Hertzian fluids [53]. Other more sophisticated closures, such as the Rogers-Young [56], may exhibit a correlation hole in the high-density regime of softly interacting fluids [27], hence giving rise to erroneous predictions. The numerical solution of the RISM scheme has been obtained by using a standard iterative Picard algorithm, on a grid of 8192 points with a mesh Δr=0.005σ. The convergence of the Picard algorithm has been improved by mixing old and new γ(r) functions with a mixing parameter of 0.9. We have verified that this choice allows for an optimization of the convergence procedure in the whole range of temperatures and densities investigated in this work.

## 3. Results and Discussion

**Figure 2:**
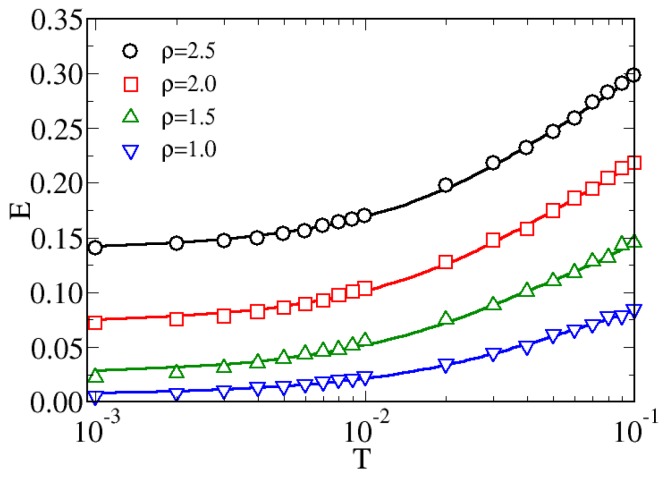
MC potential energy of per particle *E* of hard-soft dimers as a function of the temperature for various densities.

Since the formation of aggregates usually takes place in the low-temperature regime we have investigated the effect of progressively cooling the system starting from a relatively high temperature, corresponding to $T=1\times 10^{-1}$. Indeed in previous studies [31,53,54] it has been observed that Hertzian spheres are in a homogeneous fluid phase at this temperature. In addition, we have deeply investigated also the role played by the density in determining the final structure of the hard-soft dimers. In Figure 2 we show the behavior of the potential energy per particle *E* obtained from MC simulations. Starting from relatively high values for $T=1\times 10^{-1}$, the energy monotonically decreases with the temperature for all the considered densities: this effect can be clearly observed by setting a semilogarithmic scale. The *E*-dependence on *T* is smooth and continuos and no kinks are observed, unlike documented, for instance, in a previous simulation study of cluster formation in patchy colloids [57]. This suggests that the mechanism of aggregation in hard-soft dimers is different from that observed in colloidal particles with anisotropic attractive interactions, since in that case the particles usually start to self-aggregate below a threshold temperature and this mechanism involves all particles in the simulation box. Conversely, in our simulations the aggregation seems to proceed at a local scale, with small rearrangements of the particle positions with causes the smooth decrease of the energy.

**Figure 3:**
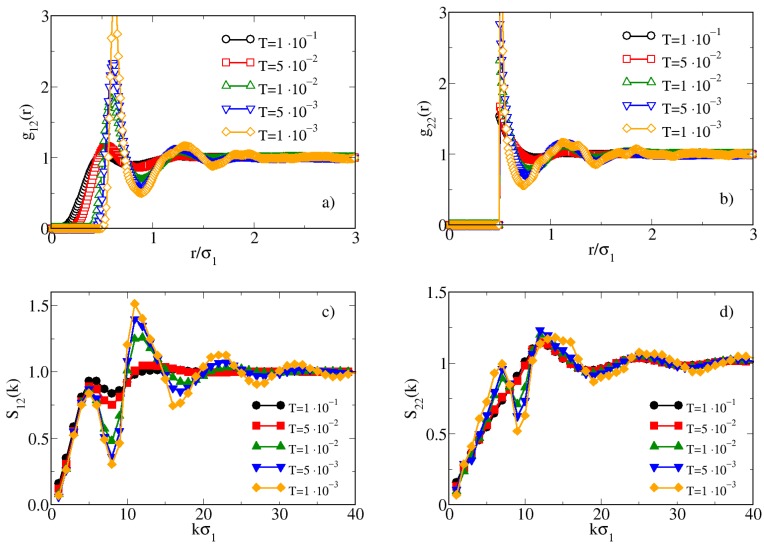
MC radial distribution functions (**a**,**b**) and structure factors (**c**,**d**) for hard-soft dimers for ρ=2.0 and various temperatures. The subscripts 1 and 2 label the hard and soft monomers, respectively.

In order to validate this conjecture we have analyzed in detail the fluid structure by calculating the site-site radial distribution functions $g_{ij}\left(r\right)$ and structure factors $S_{ij}\left(k\right)$ for a given density, high enough to allow the aggregation to take place. The results are reported in Figure 3 for ρ=2.0.

By analyzing the behavior of $g_{ij}\left(r\right)$ (top panels) we note that for $T\geq 5\times 10^{-2}$ both $g_{12}\left(r\right)$ and $g_{22}\left(r\right)$ are poorly structured and no evidence of a self-assembly can be found. On the other hand, upon cooling the system from $T=5\times 10^{-2}$ to $T=1\times 10^{-2}$, the radial distribution functions suddenly become well structured and for still lower temperatures this structuring is increasingly evident. The behavior of the structure factors $S_{ij}\left(k\right)$ provides further information on the possible appearance of aggregates in the system. Indeed, for $T<5\times 10^{-2}$ both $S_{12}\left(k\right)$ and $S_{22}\left(k\right)$ show two peaks: beside the main peak, placed at a wavevector corresponding to the sphere-sphere interparticle distance, a peak placed at lower wavevectors is found. It is known that such a feature is usually associated to the insorgence of an intermediate range order typical of a cluster fluid [58,59]. Noteworthy, the low-*k* peak is observed even for the $S_{22}\left(k\right)$, i.e., for the structure factor involving the two hard spheres of the dimers: therefore, although the site-site hard sphere interaction does not depend on the temperature, the presence of a soft interaction on the first sphere of the dimer causes a *T*-dependence also of the hard sphere. The emerging picture is compatible with a fluid structure which progressively rearranges from homogeneous to cluster fluid upon lowering the temperature, even if no attraction in the intermolecular interaction is set. This behavior qualitatively agrees with experimental results on hard-soft dimers [40,41,42] where clusters are formed by partially overlapping the soft spheres of different dimers. We will further discuss on the agreement between our model and experimental data later on.

**Figure 4:**
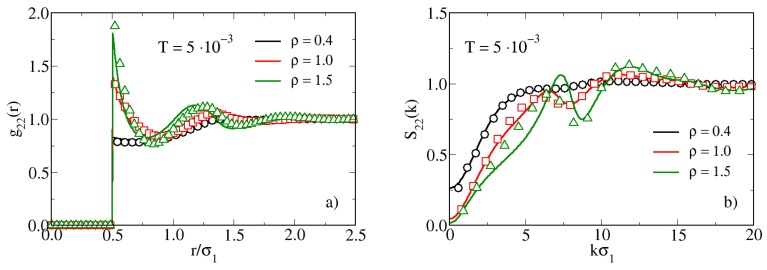
Radial distribution functions (**a**) and structure factors (**b**) for hard-soft dimers for T=0.005 and various densities, obtained from RISM theory (full lines) and MC simulations (symbols).

In order to shed light on this scenario, we make use of the RISM theory of molecular liquids which, together with the Ornstein-Zernike integral equation description of atomic fluids [55], has been already adopted in previous studies to characterize the onset of aggregates in non-homogenous fluids [60,61,62]. In Figure 4 we compare RISM and MC results for $g_{22}\left(r\right)$ [panel (a)] and $S_{22}\left(k\right)$ [panel (b)] at fixed T=0.005. The RISM theory closely follows the simulation data in reproducing the increasing height of the peak of the $g_{22}\left(r\right)$ upon increasing the density from ρ=0.4 to 1.5. The increase of this peak is clearly due to packing effects, with dimers becoming progressively closer. On the other hand, upon analyzing the behavior of $S_{22}\left(k\right)$, we observe the appearance of a double-peak structure for ρ=1.0, which becomes well defined for ρ=1.5. This feature is not barely a packing effect, but rather it suggests the insorgence of local inhomogeneities, as already observed upon cooling the system (see Figure 3). Interestingly, the RISM theory predicts the double peak in the $S_{22}\left(k\right)$ as well, even if it is slightly misplaced in comparison to MC simulations.

**Figure 5:**
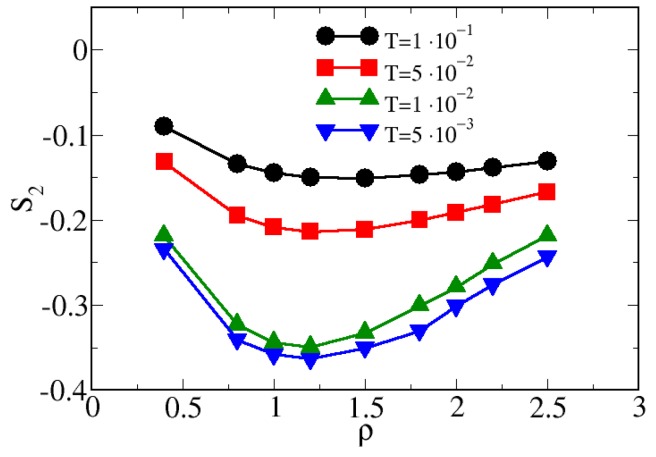
MC pair translational entropy $s_{2}$ for various temperatures as a function of the density.

The analysis of the structure of the hard-soft dimer fluid has been completed by calculating the pair translational entropy $s_{2}$, defined as [30,63,64,65]:

(8) $$s_{2}/k_{B}=-\frac{1}{2}\rho \int dr\left[g_{cm}\left(r\right)\ln g_{cm}\left(r\right)-g_{cm}\left(r\right)+1\right]\;,$$

where $g_{cm}\left(r\right)$ is the pair distribution function between the centers of mass of two dimers. This quantity effectively characterizes the degree of pair translational order existing in the fluid [66]. In Figure 5 we report the behavior of $s_{2}$ as a function of the density for different temperatures. When the system is cooled, $s_{2}$ non-monotonically depends on ρ, showing a minimum placed at ρ≈1.25. This minimum, barely sketched for $T\geq 5\times 10^{-2}$, becomes very well defined for lower temperatures. The range of temperatures where the minimum develops is the same range where the $S_{22}\left(k\right)$ shows the double-peak behavior, confirming the onset of inhomogeneities at the local scale. Furthermore, the non-monotonic behavior of $s_{2}$ is the fingerprint of a structural anomaly in the system [30]. This could suggest the existence of a reentrant melting at lower temperatures; we plan to perform a deeper investigation of this possible scenario in future works.

**Figure 6:**
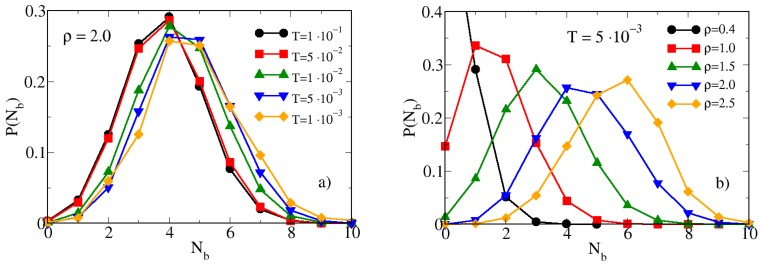
Panel (**a**) MC probability distribution of bonds between hard and soft monomers of different dimers for ρ=2.0 and various temperatures. Panel (**b**) same for T=0.005 and various densities.

The combined theoretical-simulation analysis of the fluid structure of hard-soft dimers at low temperatures is therefore compatible with the onset of local inhomogeneities. Even if the energy decays smoothly with the temperatures (see Figure 2), the inhomogeneities appear abruptly on the local scale. This confirms that in our model the onset of aggregates is quite sudden only on the local scale, as signalled by $S_{12}\left(k\right)$ and $S_{22}\left(k\right)$, since it is driven by the molecular geometry and by the softness of the potential rather than attractive interactions. In order to characterize this phenomenon we have calculated the probability distribution of bonds between hard and soft monomers of different dimers. In our approach, hard and soft monomers are considered bonded if their reciprocal distance is smaller than the abscissa $r_{min}$ of the first minimum of the $g_{12}\left(r\right)$. Therefore, since $r_{min}$ is little dependent on both temperature and density (see Figure 3 and Figure 4), in what follows we assume in all cases $r_{min}=0.9$. In Figure 6 we report the probability distributions of bonds $P(N_{b})$ between hard and soft monomers for various temperatures [panel (a)] and densities [panel (b)]. At fixed ρ=2.0 [panel (a)] and high temperatures, $P(N_{b})$ shows a peak centered at a number of bonds $N_{b}=4$. Upon cooling the system the peak becomes less sharp and for $T<1\times 10^{-}2$ the maximum of $P(N_{b})$ is found at both $N_{b}=4$ and $N_{b}=5$. Interestingly, the range of temperatures where this change is observed is the same in which the radial distribution functions become sharper and the structure factors show a double peak (see Figure 3). In order to make a more quantitative comparison, we have calculated the coordination number $N_{12}\left(r_{min}\right)$, defined as:

(9) $$N_{12}\left(r_{min}\right)=4\pi \rho \int_{0}^{r_{min}}g\left(r\right)r^{2}dr$$

which provides the average number of hard (or soft) monomers inside a shell of radius $r_{min}$ centered on a soft (or hard) given monomer. In our simulations we have obtained $N_{12}\left(r_{min}\right)=5.42,5.43,5.63,5.69$ and 5.72 for $T=1\times 10^{-1},5\times 10^{-2},1\times 10^{-2},5\times 10^{-3}$ and $1\times 10^{-3}$, respectively. The increase of the average number of neighbours of a given sphere upon cooling the system closely resembles the behavior of $P(N_{b})$ shown in Figure 6a. The effect of increasing the density at fixed T=0.005 is investigated in Figure 6b: in this case the peak of $P(N_{b})$ systematically moves towards progressively higher values of $N_{b}$, since dimers get closer and closer. It may be worth noting that no saturation effect is found, this indicating that hard and soft monomers are able to rearrange their configurations in order to minimize the occupied volume fraction when the density increases.

**Figure 7:**
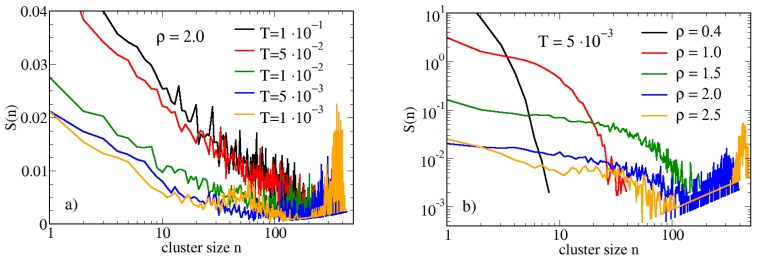
Panel (**a**) MC cluster size distribution of hard-soft dimers for ρ=2.0 and various temperatures. Panel (**b**) same for T=0.005 and various densities.

In the same range of temperatures and densities shown in Figure 6 we have calculated the cluster size distribution (CSD) of hard-soft dimers. In Figure 7 we report the CSD for various temperatures [panel (a)] and densities [panel (b)]: according to the procedure described in Ref. [67] the CSD S(n) has been normalized as follows:

(10) $$S\left(n\right)\equiv \frac{n\times P(n)}{N_{c}}$$

where *n* is the cluster size, P(n) is the probability to find a cluster of size *n* and $N_{c}$ is the total number of clusters. In order to highlight the *n*-dependence of the CSD, the x-axis is reported in a logarithmic scale. For ρ=2.0 (Figure 7a) and $T\geq 5\times 10^{-2}$ the CSD is typical of an homogeneous fluid, since it monotonically decreases with the cluster size *n* [68,69]. If the system is cooled down to $T=5\times 10^{-2}$, the CSD starts to exhibit a non-monotonic behavior, showing a peak placed towards high values of *n*. This peak, which appears for $T=1\times 10^{-2}$, becomes well defined for $T=1\times 10^{-3}$, suggesting that under these conditions a very large fraction of the dimers is bonded into a single cluster. A similar effect is observed by keeping fixed the temperature and increasing the density, as observed in Figure 7b for T=0.005: under dilute conditions, the CSD is again compatible with a homogeneous fluid, as expected. Upon increasing ρ, the shift of the peak towards high values of *n* is observed also in this case; it is worth noting the presence of a logarithmic scale on the y-axis, beside the x-axis. This can be expected, since, due to the absence of any attraction, the effects due to the density are stronger than those ascribed to the temperature. We have not investigated higher densities in order to avoid the possible appearance of solid phases.

**Figure 8:**
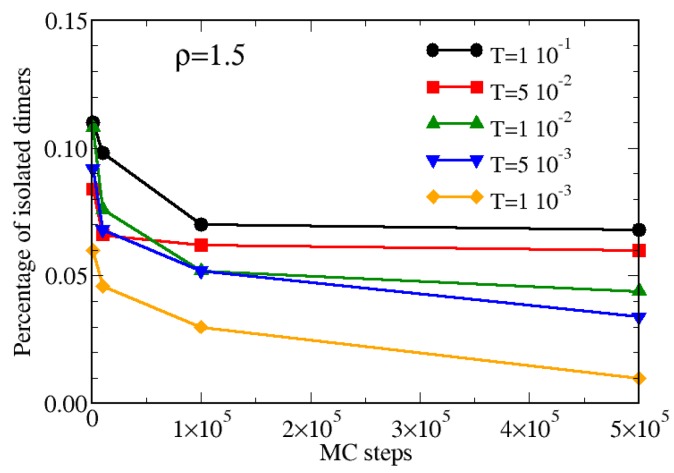
Percentage of isolated hard-soft dimers as a function of the MC steps for ρ=1.5 and various temperatures.

The analysis of the CSD allows us also to perform a qualitative comparison with experimental results on synthesized hard-soft dimers reported in Figure 2 of Ref. [40]: in particular, in that work the authors showed how the flocculation of the dimers in solution changes upon adding a steric stabilizer. Since the latter prevents a too quick self-assembly of dimers, its role can be thought as the inverse of temperature in our simulations. In analogy with the results presented in Ref. [40], we show in Figure 8 the percentage of isolated dimers as a function of the MC steps upon cooling the system for ρ=1.5. For high temperatures, after a first quick decay, the numer of isolated dimers fluctuates around a constant value which depends on the specific value of $T^{*}$, as observed in experiments when an high amount of steric stabilizer is added. Conversely, for low temperatures, the percentage of isolated dimers systematically decreases with the number of MC steps, since the system requires more time for attaining the equilibrium: this is analogous to the experimental case where no steric stabilizer is added and the self-assembly may take place, as shown in Figure 2 of Ref. [40].

The analysis of the cluster structures is completed by calculating the average cluster size (ACS), according to the formula:

(11) $$\chi \left(n\right)\equiv \frac{\sum_{n=1}^{N_{c}}n^{2}\times P\left(n\right)}{\sum_{n=1}^{N_{c}}n\times P\left(n\right)}$$

**Figure 9:**
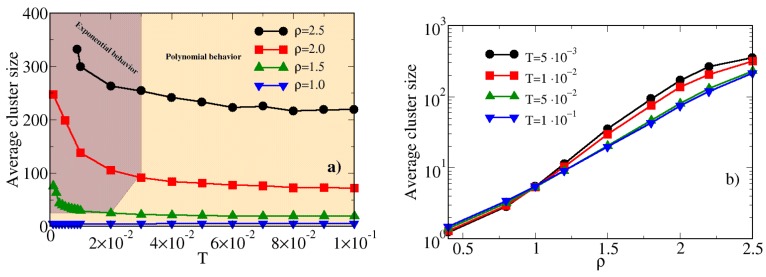
Panel (**a**) MC average cluster size of hard-soft dimers for various densities as a function of the temperature. The different curves can be fitted by using a polynomial fit or an exponential fit: the two different regimes are indicated by the orange and brown areas, respectively. Panel (**b**) MC average cluster size of hard-soft dimers for various temperatures as a function of the density.

In Figure 9a we report the ACS for hard-soft dimers for various densities as a function of the temperature. Upon decreasing the temperature, $\chi_{n}$ monotonically increases, provided that the density is high enough (namely, ρ≥1.5). Therefore, as observed also by analyzing the CSD, there is a threshold in the density, over which the clusterization may take place. If the system is too dilute, the dimers can not interact. This is a fundamental difference in comparison to systems where attraction is set, since in the latter case the particles can give rise to a large variety of structures in the low-density regime [21,24,70]. In addition, we have also verified that, even if the increase of the ACS is monotonic, the *T*-dependence of χ follows a polynomial law for $T\geq 3\times 10^{-2}$, becoming exponential for $T<3\times 10^{-2}$, provided that the density is high enough. These two different regimes are represented in Figure 9 by the orange and brown areas, respectively. The change of the *T*-dependence of χ is a further fingerprint of the development of inhomogeneities in the system; in particular, in the brown region, a tiny change of the temperature causes a remarkable increase of the ACS. A change in the behavior of the ACS is shown also in Figure 9b, where χ is reported as a function of the density for various temperatures. Interestingly, we note that for $T\geq 5\times 10^{-2}$ the ρ−dependence of χ can be fitted by a straight line, whereas for lower temperature there is a change of the concavity of the curve fitting the ACS. This change is placed at ρ≈1.25, the same value where the minimum in the $s_{2}$ is found (see Figure 5) and close to the value where the CSD suggests the existence of large clusters (namely ρ=1.5, see Figure 7).

**Figure 10:**
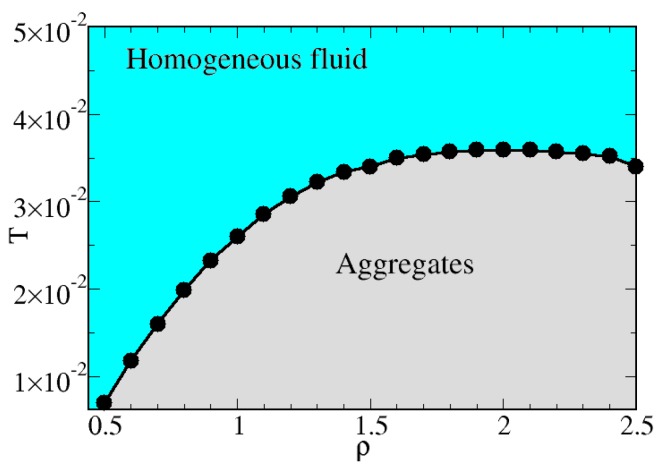
RISM predictions for the structural behavior of hard-soft dimers. The cyan zone is separated from the grey area by a border line which identify the appearance of the low-*k* peak in the $S_{22}\left(k\right)$. The region corresponding to the solid phase (expected for $T<10^{-3}$) is not reported.

Finally, after collecting structural and thermodynamics observations, we have characterized the behavior of the hard-soft dimers in the fluid phase by using the RISM theory. Specifically, in Figure 10 we report the theoretical predictions for the first appearance of the low−k peak in the $S_{22}\left(k\right)$. The predicted values give rise to a border line which separates a region (cyan in the figure) in the temperature-density diagram where a homogeneous fluid is observed from another region (grey in the figure) where a locally non-homogeneous cluster fluid exists. The advantage of using the RISM approach to characterize these two regimes relies on both the accuracy of the theory in reproducing the structure of the hard-soft dimer fluid (see Figure 4) and on the possibility to perform a fine spanning of different thermodynamic conditions, needed to accurately observe the delopment of the low−k peak. For low densities, the system needs to be significantly cooled in order to observe the appearance of inhomogeneities; then, upon increasing the density, the temperatures where clusters are expected increases in turn, until it ranges between 0.03 and 0.04. Noteworty, this temperature regime closely match the previous indications on the onset of aggregates provided by cluster analysis, bonds distributions and correlation functions.

## 4. Conclusions

In this work we have investigated the onset of local inhomogeneities in a simple model of hard-soft dimers without any attractive interaction. In our approach two hard spheres belonging to different dimers experience a hard-core interaction, whereas two soft spheres interact via a soft Hertzian potential. The cross correlations are also Hertzian, even if with a lower degree of softness. By performing Monte Carlo (MC) simulations and integral equation calculations, based on the Reference Interaction Site Model (RISM) theory we have provided evidence of inhomogeneities in the system, which suggest the onset of aggregates. Such an evidence is based on the analysis of radial distribution functions, structure factors, pair correlation entropy, cluster size distribution, average cluster size and bonds distribution. Noteworthy, RISM theory and MC simulations agree in documenting the existence of a temperature range [$5\times 10^{-2}$–$1\times 10^{-2}$] and a density range [1.25–1.5] where these inhomogeneities take place. In particular, the theory well catches the development of a low-*k* peak in the structure factor observed in simulations; this feature is usually associated with the development of an intermediate range order (and therefore of structural inhomogeneities) in the system. As a futher interesting consideration, a T—dependence is found also for the hard-core interactions, testified in particular by the development of a low-*k* peak in the corresponding structure factor. The existence of a T—dependence for a simple hard-sphere potential (even if induced by the softness of the other interactions) constitutes one of the main novelties of our study, since, to the best of our knowledge, it has not been documented so far in the large variety of simulation studies of repulsive potentials performed in the literature. These indications set our model as a simple and useful tool to investigate the onset of inhomogeneities in systems where the attractive interactions are negligible. Furthermore, the importance of soft potentials in driving the final structure of colloidal molecules is highlighted, paving the way for a more extended and systematic investigation of the proposed models for studying these systems.
